# *CD44 *rs13347 C>T polymorphism predicts breast cancer risk and prognosis in Chinese populations

**DOI:** 10.1186/bcr3225

**Published:** 2012-07-12

**Authors:** Lan Jiang, Jieqiong Deng, Xun Zhu, Jian Zheng, Yonghe You, Na Li, Hongchun Wu, Jiachun Lu, Yifeng Zhou

**Affiliations:** 1 Laboratory of Cancer Molecular Genetics, Medical College of Soochow University, Suzhou 215123, China; 2 Department of General Surgery, the Second Affiliated Hospital of Soochow University, San Xiang Road No. 1055, Suzhou 215004, China; 3 The Institute for Chemical Carcinogenesis, the State Key Lab of Respiratory Disease, Guangzhou Medical College, 195 Dongfengxi Road, Guangzhou 510182, China

## Abstract

**Introduction:**

It has been demonstrated that the interplay of adhesion molecule CD44 and its ligands can regulate cancer cell proliferation, migration and invasion, as well as tumor-associated angiogenesis and is related to breast cancer patient survival. In this two-stage, case control study, we determined whether common functional tagSNPs (single nucleotide polymorphisms) are associated with breast cancer risk and prognosis.

**Methods:**

Five tagSNPs of *CD44 *(rs10836347C>T, rs13347C>T, rs1425802A>G, rs11821102G>A, rs713330T>C) were selected and genotyped in 1,853 breast cancer patients and 1,992 healthy control subjects in Eastern and Southern populations. Potential function of rs13347C>T and association between this variation and breast cancer were further studied.

**Results:**

Compared with the most common rs13347CC genotype, variant genotypes (CT and TT) increased an individual's susceptibility to breast cancer, especially in estrogen receptor (ER) negative patients (odds ratio (OR) = 1.37, 95%CI = 1.17 to 1.59 for ER positive patients; OR = 2.37, 95% CI = 2.00 to 2.80 for ER negative patients). We also found that rs13347CT+ TT genotypes predicts lower five-year survival rate (hazard ratio (HR) = 1.85, 95% CI = 1.09 to 3.15, *P *= 0.023), with the lowest survival probability in ER negative T allele carriers. Furthermore, our reporter assay findings, although preliminary and rather modest, showed that miR-509-3p may suppress CD44 expression more strongly in C allele carriers than T allele carriers (*P *< 0.01). Similarly, rs13347 variant genotypes (CT and TT) carriers were shown to have more CD44 expression than CC carriers in both immunohistochemistry (*P *< 0.001) and western blotting (*P *= 0.001) results.

**Conclusion:**

These findings suggest that *CD44 *rs13347C>T polymorphism may affect breast cancer development and prognosis by increasing CD44 expression.

## Introduction

With gradually increasing incidence and mortality, breast cancer refers to malignant tumor originating from breast tissue, most commonly from the inner lining of milk ducts or the lobules that supply the ducts with milk [1]. Excluding cervical cancer, it is the most frequent cancer killer of middle-aged women [2]. Recent studies have established some etiologic factor for breast cancer, such as ionizing radiation [3], alcohol consumption [4], high-fat diets [5], oral contraceptives and use of hormones in treatment of certain diseases [6]. Excluding these environmental factors, genetic variations also play an important role in an individual's risk of developing breast cancer [7].

Compelling evidence has demonstrated that breast cancers contain few phenotypically distinct cells, known as breast cancer-initiating cells (BCICs), which account for primary and metastatic tumor growth [8,9]. BCICs can be distinguished from other breast cancer cells by the expression of so-called CIC-markers that play a vital role in BCIC maintenance and activity [10]. CD44 is one of the well known markers of BCIC, which may contribute not only to drug and radiation resistance of BCIC but also preparation of the pre-metastatic niche [11].

By cell-cell and cell-extracellular matrix adhesive interactions, CD44 participates in some fundamental biological processes, including lymphocyte homing, cell migration, haematopoiesis, inflammation, wound healing, embryonal development and apoptosis [12]. Besides, CD44 also plays an indispensable role in tumor pathology, involved in cell differentiation, invasion and metastasis [13-15]. Also, some studies reported strong association between CD44 expression and breast cancer aggressiveness [16,17]. Correspondingly, some studies have recently indicated qualitative and quantitative changes in CD44 expression in breast cancer [18].

Since expression of CD44 is closely related to development of breast cancer and genetic variations in certain genes may affect their expression [19], we hypothesize that variations in *CD44 *that can theoretically affect its protein expression may be associated with varying risk and prognosis of breast cancer. In this study, five eligible tag single nucleotide polymorphisms (tagSNPs) of *CD44 *gene were selected from the Genbank dbSNP database to evaluate the contribution of detected polymorphisms to risk of developing breast cancer. One of them is an A/G polymorphism (rs1425802) in the promoter region, the conversion from A to G cause loss of an Nkx-2 binding site, which may theoretically affect the *CD44 *transcriptional activity. Another T/C (rs713330) polymorphism in the intron was linkage disequilibrium with the non-synonymous rs9666607 G>A polymorphism, which may change the 417 amino acid from Arg to Lys. The other three polymorphisms (rs13347C/T, rs10836347C/T, rs11821102G/A) all locate in the 3'UTR of *CD44*, each of which can cause a change in the binding ability of certain MicroRNA between the two different alleles. Only one published research article has investigated polymorphisms in CD44 exon2 and breast cancer [20]; however, no study has investigated the role of tagSNPs that cover all common polymorphisms in breast cancer risk. So, we carried out a hospital-based, case-control study including 1,853 breast cancer patients and 1,992 cancer free controls to investigate the contribution of the five polymorphisms of *CD44 *to susceptibility to and prognosis of breast cancer.

## Materials and methods

### Study subjects for case-control and follow-up study

All subjects in the case-control study were ethnically homogenous Han Chinese derived from the Eastern Chinese population or Southern Chinese population. In the Eastern Chinese population, patients with newly diagnosed breast cancer (*n *= 1,049) were consecutively recruited from the First Affiliate Hospital of Soochow University (Suzhou) during March 2001 to May 2009. All the eligible patients diagnosed at the hospital during the study period were recruited, with a response rate of 89%. Patients were recruited from Suzhou city and its surrounding regions, and there were no age, stage and histology restrictions. Population controls (*n *= 1,157) were cancer-free people living in Suzhou region; they were selected from a nutritional survey conducted in the same period as the cases were collected [21]. In the Southern Chinese population, breast cancer cases (*n *= 804) were recruited from the Tumor Hospitals affiliated with Guangzhou Medical College between 2002 and 2009 with a response rate of 91%. Cancer-free controls (*n *= 835) were randomly selected from a pool of 5,000 individuals who participated in a community-based screening program for a health checkup conducted in Guangdong province during the same time period when the cases were recruited [22]. The pathological type and tumor staging were evaluated according to the 2002 American Joint Committee on Cancer staging system. The clinical features of the patients are summarized in Additional file 1, Table S1. The patients were frequency matched to controls on age. In Suzhou center, the average age was 49 years (range 21 to 79) for case patients, and 49 years (range 20 to 81) for control subjects (*P *= 0.57); in Guangzhou center, the average age was 48 years (range 14 to 88) for case patients, and 47 years (range 17 to 79) for control subjects (*P *= 0.60)

For the five-year survival rate study, 566 breast cancer patients with relatively complete clinical information from the First Affiliate Hospital of Soochow University were followed up as the discovery set. Similarly, 331 patients from tumor hospitals affiliated with Guangzhou Medical College were involved in the validation set. Patients were followed-up by telephone calls every three months and survival time was calculated from the date when patients first received confirmed diagnoses until the date of the last follow-up or death. Dates of death were obtained from inpatient and outpatient records or from the patients' families through telephone follow-up. Clinical features of the subjects for the follow-up studies were shown in Additional file 2, Table S2.

At recruitment, informed consent was obtained from each subject. This study was approved by the Medical Ethics Committee of The First Affiliate Hospital of Soochow University and Tumor Hospitals affiliated with Guangzhou Medical College.

### TagSNPs selection

Bioinformatics analysis with Haploview software 4.2 (Mark Daly's lab of Broad Institute, Cambridge, MA, Britain) was performed to analyze the haplotype block based on the CHB (Chinese Han Beijing) population data of HapMap (HapMap Data Rel 27 PhaseII +III, Feb 09, on NCBI B36 assembly, dbSNP b126 (International HapMap Project). Six tagSNPs were found to cover all the potential functional common SNPs (MAF > 0.05) in the *CD44 *gene: rs8193, rs11821102, rs10836347 and rs13347 in the 3'UTR, rs1425802 in the promoter and rs9666607 in exon region (Additional file 3, Figure S1). Among them, rs8193 and rs13347 were in high linkage disequilibrium (LD) (*D' *= 1.0, *r^2 ^*= 0.527), so the selection of rs13347 is enough to represent the two SNPs. Besides, due to the difficulty in genotyping rs9666607 by MALDI-TOF method, we chose rs713330, which is in complete LD with rs9666607 (*D' *= 1.0, *r^2 ^*= 1) to replace it.

### Genotyping analysis

Genomic DNA was isolated from the peripheral blood lymphocytes of the study subjects. MassArray (Sequenom, San Diego, CA, USA) was used for genotyping all markers using allele-specific MALDI-TOF mass spectrometry [23]. Primers and multiplex reactions were designed using the RealSNP.com Website. All breast cancer patients and healthy controls in Suzhou center were genotyped for rs10836347, rs13347, rs1425802, rs11821102 and rs713330 polymorphisms. Patients and controls from Guangzhou center were genotyped only for the polymorphism rs13347 to warrant the results of Suzhou.

### Construction of *CD44 *3'UTR luciferase reporter plasmids

Based on bioinformatics analysis, *CD44 *rs13347 C not T is predicted to lie in a hsa-mir-509-3p binding site. Therefore, we hypothesized that hsa-mir-509-3p would bind tightly to *CD44 *mRNA transcripts containing the C allele, negatively regulating *CD44 *expression. To test this hypothesis, the T and C allelic reporter constructs were respectively prepared by amplifying a 362-bp CD44 3'UTR region from subjects homozygous for the T and C allele, including the artificial XhoI and NotI enzyme restriction sites with forward primer 5'-ATCG CTCGAG GGCCATTGTCAACGGAGA-3' and reverse primer 5'- ATGC GCGGCCGC CAGGCTTGAAATATGGATTCG-3'. The amplified fragments were then cleaved with the XhoI and NotI enzymes (New England BioLabs, Ipswich, MA, USA). The psiCHECK2 vector (Promega, Madison, WI, USA) was also cleaved with the XhoI and NotI enzymes, and the above-prepared fragment and psiCHECK2 vector were then ligated by T4 DNA ligase (New England BioLabs). The two constructs were sequenced to confirm the allele, the orientation and integrity of each insert.

### Transient transfections and luciferase assays

293T or MCF-7 cells were maintained in Dulbecco's modified Eagle's medium with high glucose (Gibco, Los Angeles, California, USA) supplemented with 10% heat-inactivated fetal bovine serum (Gibco) and 50 μg/ml streptomycin (Gibco) at a 37°C incubator supplemented with 5% CO2. Cells were seeded at 1 × 10^5 ^cells per well in 24-well plates (BD Biosciences, Bedford, MA, USA). Sixteen hours after the plating, cells were transfected by Lipofectamin 2000 (Invitrogen, Carlsbad, California, USA) according to the manufacturer's suggestion. In each well, 800 ng psiCHECK-2-*CD44*-3'UTR vectors were co-transfected with 50 pmol hsa-mir-509-3p mimics (Ambion, Austin, TX, USA) and 40 pmol hsa-mir-509-3p inhibitor accordingly. The hsa-mir-509-3p inhibitor is single-stranded RNA molecules, which can specifically knock-down endogenous hsa-mir-509-3p. In addition, 100 pmol Negative Control #1 from Ambion was in every transfection experiment. There are six replicates for each group and the experiment is repeated at least three times. Twenty-four hours after transfection, cells were harvested by passive adding of 100 μl buffer. Renilla luciferase activities in cell lysate were measured with the Dual-Luciferase Reporter assay system (Promega) in TD-20/20 luminometer (Turner Biosystems, Sunnyvale, CA, USA) and were normalized with the firefly luciferase activities.

### Western blotting analysis

To analyze the correlation between rs13347 C>T polymorphism in 3' UTR of *CD44 *and the protein expression levels in breast cancer tissues, Western blotting assays were performed. Generally, 39 breast cancer tissues were homogenized in 800 μl detergent lysis buffer and then the tissue homogenates were centrifuged at 12,000 g for 15 minutes to get the supernatant. Sixty micrograms of total proteins (the supernatant) were run on a SDS-polyacrylamide gel electrophoresis (SDS-PAGE) and transferred to PVDF (Millipore, Billerica, MA, USA). The membrane was blocked with 5% milk in tris-buffered saline (TBS) with 0.05% Tween-20 for one hour at room temperature with constant agitation. The polyclonal antibody against CD44 and the monoclonal antibody against GAPDH were both purchased from Santa Cruz Biotechnology (Santa Cruz, CA, USA). The membranes were incubated overnight at 4°C with the primary antibody diluted 1:1,000 and the proteins were detected with a Phototope-horseradish peroxidase Western blot detection kit (Cell Signaling Technology, Danvers, MA, USA). The CD44 protein expression levels were normalized to that of GAPDH by calculating the relative expression levels.

### Immunohistochemistry analysis

After screening hematoxylin and eosin-stained slides for optimal tumor content, we constructed tissue slides. Cores were taken from each formalin-fixed, paraffin-embedded breast cancer samples by using punch cores that measured 0.8 mm in greatest dimension from the center of tumor foci. Immunohistochemistry for CD44 was performed by using the avidin-biotin complex method (ABC; Vector Laboratories, Burlingame, CA, USA), including heat-induced antigen-retrieval procedures. Primary antibodies were mouse antihuman monoclonal antibodies combined with CD44 (1:200; Santa Cruz Biotechnology,). The components of the Envision-plus detection system (EnVision+/HRP/Mo; Dako, Carpinteria, CA, USA) were applied. Reaction products were visualized by incubation with 3, 3'-diaminobenzidine. Negative controls were treated identically but with the primary antibody omitted. The images of stained slides were obtained and evaluated by experienced pathologists. The percentage of positive tumor cells was determined and graded (0 to 5): 0% (0), 1 to 20% (1), 21 to 40% (2), 41 to 60% (3), 61 to 80% (4) and > 81% (5) [24].

### Statistical analysis

Two-sided chi-square tests were used to assess differences in the distributions of age, menstrual history, body mass index (BMI) and family history of breast cancer between cases and controls as well as the allele and genotypes. The Hardy-Weinberg equilibrium (HWE) was tested by a goodness-of-fit chi-square test to compare the expected genotype frequencies with observed genotype frequencies (*p^2 ^+ 2pq + q^2 ^= 1*) in cancer-free controls. The association between case-control status and each SNP, measured by the odds ratio (OR) and its corresponding 95% confidence interval (CI), was estimated using an unconditional logistic regression model, with and without adjustment for age, BMI and family history of cancer. Logistic regression modeling was also used for the trend test [25,26]. The data were further stratified by age, age at menarche (years), menstrual history, BMI, pathological type, stage, estrogen receptor status, progesterone receptor status and family history of cancer to evaluate the stratum variable-related ORs among the *CD44 *genotypes. Homogeneity among stratum variable related ORs was tested [25]. The associations between overall survival time and demographic and clinical characteristics were estimated using the Kaplan-Meier method and Log-rank test by SAS. The effect modifications by these characteristics and the effects of SNPs on death risk in patients with breast cancer were assessed using the Wald test in the multivariate Cox proportional hazards regression models after adjusting for the confounders. The proportional hazards assumption was examined by testing interactions between the genotypes and time (all *P-*value > 0.05). The differences in the luciferase reporter activity, normalized expression values and protein level in cancer tissue of *CD44 *(Western blot ratio and IHC scores) between each allele were analyzed by Kruskal-Wallis one way ANOVA. The tests were all two-sided and analyzed using the SAS software (version 9.1; SAS Institute, Cary, NC, USA). *P *< 0.05 was considered statistically significant.

## Results

### Genotypes and risk of breast cancer

The association of breast cancer with rs13347C>T was performed by two independent laboratories at Soochow University and Guangzhou Medical College in Eastern (1,049 cases and 1,157 controls, Jiangsu Province) and Southern (804 cases and 835 controls, Guangdong Province) Chinese populations. The polymorphisms rs10836347, rs1425802, rs11821102 and rs713330 were only genotyped in the Suzhou population (1,049 cases and 1,157 controls) (Additional file 4, Figure S2). Genotypes were confirmed by direct sequencing (Additional file 5, Figure S3). The observed genotype frequencies of the four polymorphisms in controls conformed to the HWE (*P *= 0.84 for rs13347, 0.97 for rs10836347, 0.55 for rs1425802, 0.22 for rs11821102, *P *= 0.39 for rs713330 in the Eastern population; and *P *= 0.89 for rs13347 in the Southern population, respectively). Genotyping results showed that only rs13347 was statistically, significantly associated with breast cancer in both Eastern and Southern Chinese populations (Table 1). In the Eastern Chinese population, the frequency of the rs13347 TT and CT genotype was significantly higher in patients with breast cancer (*P_trend _*< 10^-5^) compared to the healthy controls. The adjusted OR of carrying the rs13347 CT and TT genotype in Suzhou cancer patient groups were 1.69 and 2.22, respectively, compared with the rs13347 CC genotype. The association was confirmed in the Southern population where the odds of carrying the rs13347 CT and TT genotype in cancer patient groups were 1.61 (95% CI = 1.31 to 1.98) and 2.25 (95% CI = 1.51 to 3.35), respectively, compared with the rs13347 CC genotype (*P_trend _*< 10^-5^).

**Table 1:** Associations between CD44 genotypes and breast cancer risk.

	Controls (No, %)	Breast cancer patients (No, %)	OR*^a ^*(95% CI)	*P-*value*^b^*
**Discovery Set**	***N *= 1,157**	***N *= 1,049**		

rs13347 C>T				
CC	654 (56.5)	451 (43.0)	1.00 (reference)	
CT	430 (37.2)	484 (46.1)	1.69 (1.40 to 2.04)	< 10^-5^
TT	73 (6.3)	114 (10.9)	2.22 (1.59 to 3.10)	
CT+TT	503 (43.5)	598 (57.0)	1.77 (1.48 to 2.12)	
C	1,738 (75.1)	1,386 (66.1)	1.00 (reference)	
T	576 (24.9)	712 (33.9)	1.57 (1.37 to 1.80)	
rs10836347 C>T				
CC	995 (86)	906 (86.4)	1.00 (reference)	
CT	156 (13.5)	139 (13.2)	0.98 (0.76 to 1.27)	
TT	6 (0.5)	4 (0.4)	0.66 (0.18 to 2.43)	0.743
CT+TT	162 (14)	143 (13.6)	0.97 (0.75 to 1.25)	
C	2,146 (92.7)	1,951 (93.0)	1.00 (reference)	
T	168 (7.3)	147 (7.0)	0.96 (0.75 to 1.22)	
rs1425802 A>G				
AA	353 (30.5)	316 (30.1)	1.00 (reference)	
AG	563 (48.7)	513 (48.9)	1.04 (0.85 to 1.27)	
GG	241 (20.8)	220 (21.0)	1.06 (0.82 to 1.36)	0.861
AG+GG	804 (69.5)	733 (69.9)	1.04 (0.86 to 1.27)	
A	1,269 (54.8)	1,145 (54.6)	1.00 (reference)	
G	1,045 (45.2)	953 (45.4)	1.03 (0.91 to 1.17)	
rs11821102 G>A				
GG	997 (86.2)	912 (86.9)	1.00 (reference)	
AG	151 (13)	125 (12.0)	0.85 (0.65 to 1.12)	
AA	9 (0.8)	12 (1.1)	1.76 (0.70 to 4.470	0.802
AG+AA	160 (13.8)	137 (13.1)	0.90 (0.69 to 1.16)	
A	169 (7.3)	149 (7.1)	1.00 (reference)	
G	2,145 (92.7)	1,949 (92.9)	0.95 (0.74 to 1.21)	
rs713330 T>C				
TT	950 (82.1)	865 (82.5)	1.00 (reference)	
CT	194 (16.8)	172 (16.4)	0.97 (0.77 to 1.23)	
CC	13 (1.1)	12 (1.1)	1.01 (0.43 to 2.38)	0.853
CT+CC	207 (17.9)	184 (17.5)	0.98 (0.78 to 1.22)	
T	2,094 (90.5)	1,902 (90.7)	1.00 (reference)	
C	220 (9.5)	196 (9.3)	0.98 (0.80 to 1.21)	

**Validation Set**	***N *= 835**	***N *= 804**		

rs13347 C>T				
CC	492 (58.9)	362 (45.0)	1.00 (reference)	
CT	297 (35.6)	366 (45.5)	1.61 (1.31 to 1.98)	
TT	46 (5.5)	76 (9.5)	2.25 (1.51 to 3.35)	< 10^-5^
CT+TT	343 (41.1)	442 (55.0)	1.69 (1.39 to 2.07)	

**Pooled Analysis**	***N *= 1,992**	***N *= 1,853**		

rs13347 C>T				
CC	1,146 (57.5)	813 (43.9)	1.00 (reference)	
CT	727 (36.5)	850 (45.9)	1.64 (1.43 to 1.89)	
TT	119 (6.0)	190 (10.2)	2.17 (1.68 to 2.80)	< 10^-5^
CT+TT	1,886 (42.5)	1,040 (56.1)	1.72 (1.51 to 1.96)	

*^a^*Data were calculated by unconditional logistic regression, adjusted for age, BMI and family history of breast cancer. *^b^*Tests for trend of odds were two-sided and were based on likelihood ratio tests assuming a multiplicative model.

### Stratification analysis of *CD44 *rs13347 genotypes and risk of breast cancer

**Figure 1:**
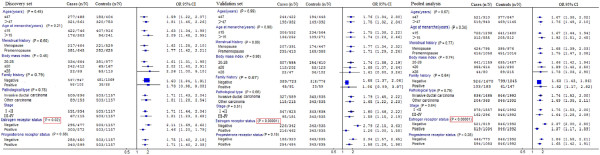
**Stratification analysis of CD44 rs13347C>T polymorphism on breast cancer risk**. ORs were adjusted for age in a logistic regression model. *P-*value of the test for multiplicative interaction between stratum-related variables and *CD44 *rs13347C>T genotypes (n, the number of CT and TT genotypes; N, the number of CC, CT and TT genotypes).

The risk of breast cancer related to *CD44 rs13347 *genotypes were further examined with stratification by age, age at menarche, menstrual history, BMI and family history of breast cancer, pathological type, clinical stage, estrogen receptor status and progesterone receptor status. As shown in Figure 1, we observed significant difference in the genotype frequency between ER-negative patients and ER-positive patients (*P *< 10^-5^). Compared with the CC genotype, the T allele carriers (CT+TT) had 2.37-fold increased risk of developing breast cancer in ER-negative patients. As for the ER-positive patients, the increased risk of CT+TT is only 1.37-fold. However, there were no differences in other subgroups.

### Regulation effects of hsa-mir-509-3p on *CD44 *3'UTR translation efficiency

**Figure 2:**
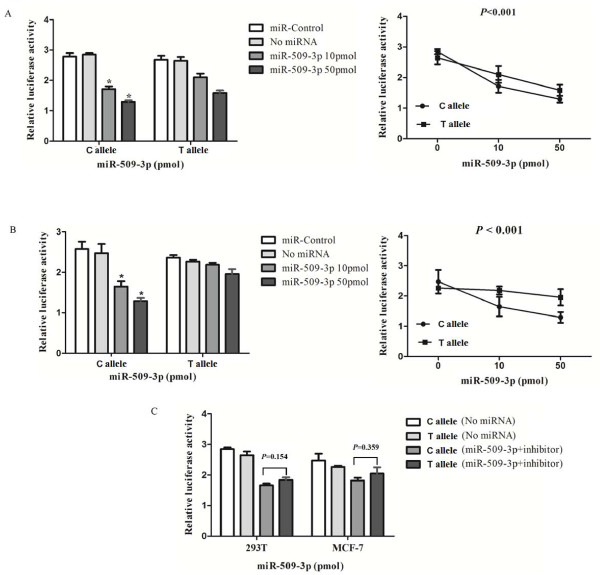
**Reporter gene expression assays modulated by hsa-mir-509-3p with constructs containing 362-bp of CD44 3'UTR**. Representative graph of luciferase activity of variant allele on luciferase reporter genes bearing 3' UTR segments from Human *CD44 *in 293T **(A) **and MCF-7 cells **(B)**. Results are shown as percentage relative to luciferase activity (Renilla luciferase activity was measured and normalized to Firefly luciferase). **(C) **Relative luciferase activity of the psiCHECK-2-*CD44*-3'UTR-C-allele and psiCHECK-2-*CD44*-T-allele constructs co-transfected with 40 pmol hsa-mir-509-3p and inhibitor. Assay was performed in 293T and MCF-7 cells. Six replicates for each group and the experiment repeated at least three times. Data are mean ± SE. *P < 0.01 compared with C allele.

Compared with the psiCHECK-2-*CD44*-3'UTR-rs13347 T, the translation of Renilla luciferase of psiCHECK-2-*CD44*-3'UTR-rs13347 C was significantly reduced in the presence of hsa-mir-509-3p in a concentration-dependent manner (*P *< 0.001), which distinguished the magnitude of the effects of hsa-mir-509-3p on the transcription of different alleles in 293T cells (Figure 2A). The same experiments were repeated in MCF-7 cells and similar results were obtained (Figure 2B). When psiCHECK-2-*CD44*-3'UTR with 50 pmol hsa-mir-509-3p and its corresponding inhibitor were cotransfected into 293T and MCF-7 cells separately, there appeared no significant difference in luciferase activity between the two recombinants (Figure 2C). These results suggest that, indeed, hsa-mir-509-3p can binds and negatively regulate the transcription of *CD44 *in the presence of rs13347 C allele.

### Effects of *CD44 *rs13347C>T variation on CD44 protein levels

**Figure 3:**
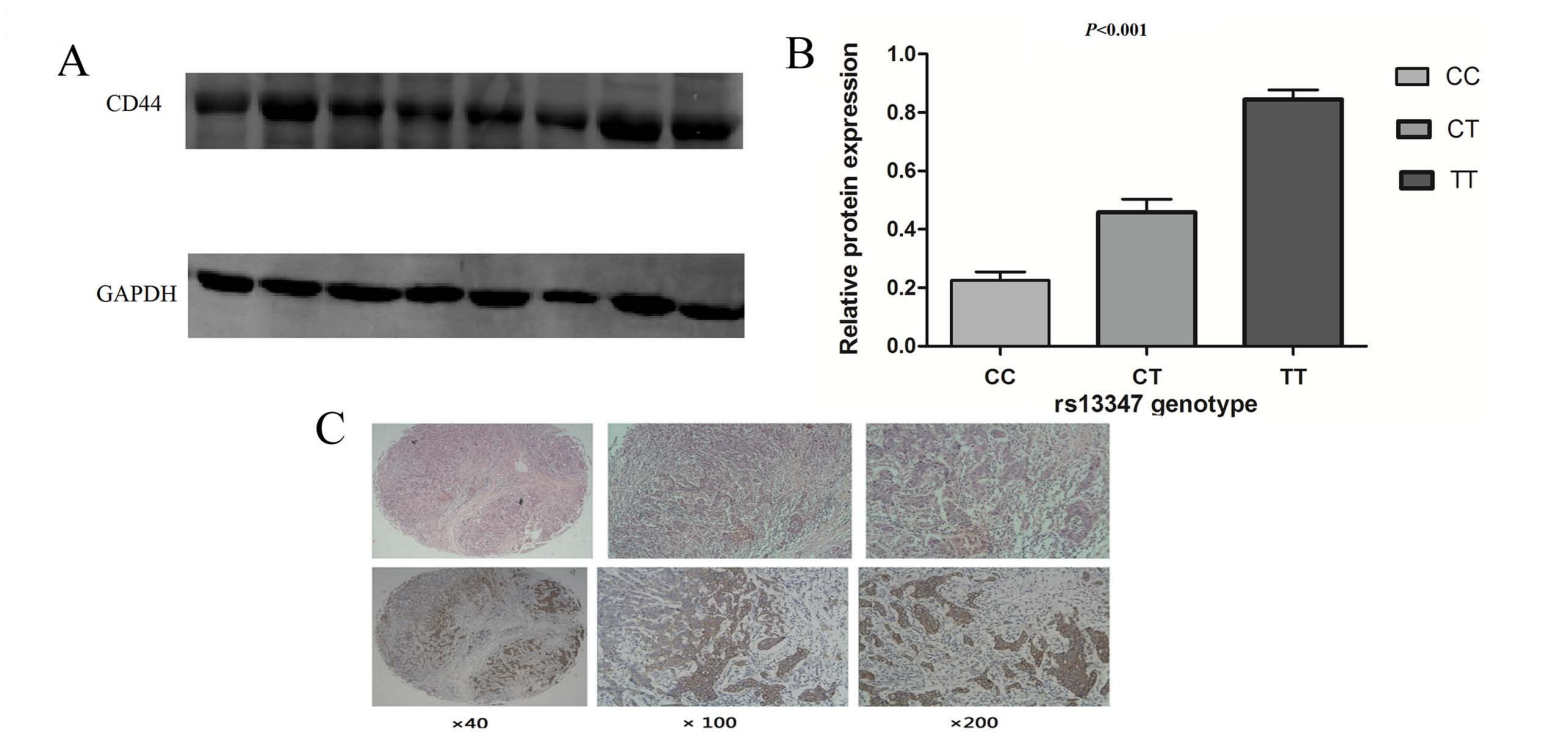
**Association between the CD44 rs13347C>T polymorphism and the CD44 protein expression**. **(A) **CD44 protein levels in 39 breast cancer tissues from individuals who carried different rs13347 genotypes. The CD44 protein expression levels were normalized to that of GAPDH by calculating the relative expression levels. **(B) **Analysis of protein levels in 39 breast cancer tissues from individuals who carried different genotypes. **(C) **Immunohistochemistry analysis of CD44 protein expression levels in breast cancer tissues. HE staining (above) and CD44 antibody staining (below) (SP, ×40, ×100, ×200).

As shown in Figure 3 and Additional file 6, Table S3, we collected 39 tumor tissues from the untreated breast cancer patients with different genotypes and found that the levels of CD44 protein of seven cases carrying the TT genotype (0.838 ± 0.127) and 17 cases carrying the TC genotype (0.465 ± 0.243) were significantly higher than that of other 15 cases carrying the CC genotype (0.238 ± 0.067) (ANOVA test: *P *< 0.001).

To confirm the results of Western blotting, we further performed the IHC study in 31 breast cancer tissues to verify association between expression level of CD44 protein and rs13347C >T *in vivo *(Figure 3C and Additional file 7, Table S4). CD44 protein expression levels in breast cancer tissues of 15 patients carrying the CC genotype were significantly lower than that in 12 patients carrying the CT or 4 patients carrying TT genotype (Kruskal Wallis Test: *P *= 0.003).

### *CD44 *rs13347C>T variation and five-year survival of breast cancer patients

The demographic and clinical characteristics of breast cancer patients in the survival discovery and validation sets are summarized in Additional file 2, Table S2. In the discovery set, the mean age was 48 years, among them, 63 (11.1%) patients died of breast cancer, 269 (47.5%) were ER negative, 242 (42.8%) were PR negative. In the validation set with the same mean age 48, 62 (18.7%) patients died of breast cancer, 139 (42.0%) were ER negative, 133 (40.2%) were PR negative. The five-year survival rates in the two sets were 88.9% and 81.3%, respectively. The Kaplan-Meier analysis, Log-rank test and univariate Cox analysis revealed that breast cancer patients that are ER or PR positive have a significantly decreased death risk (*P *= 0.0017 and *P *= 0.002, respectively). There were no significant effects of other characteristics.

Multivariate proportional hazards regression models and the Log-rank test revealed that, when compared with the rs13347 CC genotype, the rs13347 CT+TT genotypes were associated with poor survival (adjusted HR = 1.849 and *P *= 0.0233) and a lower survival probability (Log-rank *P *= 0.0211) (Table 2).

**Table 2:** Associations between CD44 genotypes and five-year survival of breast cancer

SNPs	Hazard ratio (95% CI)	P*	Breast cancer patients	Death	Log-rank *P*
***Discovery Set***			***N *= 566**	***N *= 63**	
rs10836347 C>T					
CC	1.00 (Reference)		481 (85.0)	55	
CT+TT	0.89 (0.42 to 1.88)	0.753	85 (15.0)	8	0.5854
rs1425802 A>G					
AA	1.00 (Reference)		164 (29.0)	24	
AG	0.63 (0.37 to 1.09)	0.102	276 (48.8)	28	0.2274
GG	0.73 (0.51 to 1.05)	0.091	126 (22.2)	11	
AG+GG	0.61 (0.36 to 1.01)	0.056	402 (71.0)	39	0.0951
rs11821102 G>A					
GG	1.00 (Reference)		484 (85.5)	54	
AG+AA	0.93 (0.45 to 1.91)	0.845	82 (14.5)	9	0.973
rs713330 T>C					
TT	1.00 (Reference)		468 (82.7)	51	
CT+CC	1.14 (0.59 to 2.20)	0.676	98 (17.3)	12	0.6883
rs13347 C>T					
CC	1.00 (Reference)		255 (45.1)	20	
CT	1.36 (0.75 to 2.47)	0.3189	223 (39.4)	23	0.0004
TT	3.18 (1.71 to 5.91)	0.0003	88 (15.5)	20	
CT+TT	1.85 (1.09 to 3.15)	0.0233	311 (54.9)	43	0.0211

***Validation Set***			***N *= 331**	***N *= 62**	

rs13347 C>T					
CC	1.00 (Reference)		200 (60.4)	26	
CT	2.10 (1.21 to 3.65)	0.0081	100 (30.2)	25	0.0012
TT	3.14 (1.55 to 6.38)	0.0015	31 (9.4)	11	
CT+TT	2.34 (1.41 to 3.88)	0.0010	131 (39.6)	36	0.0007

***Pooled Analysis***			***N *= 897**	***N *= 125**	

rs13347 C>T					
CC	1.00 (Reference)		455 (50.7)	46	
CT	1.54 (1.02 to 2.30)	0.0378	323 (36.0)	48	< 0.0001
TT	2.84 (1.80 to 4.48)	< 0.0001	119 (13.3)	31	
CT+TT	1.87 (1.30 to 2.70)	0.0007	442 (49.3)	79	0.0006

*The Cox regression analysis was adjusted for age, BMI, family history, TNM stage, estrogen receptor status, progesterone receptor status, pathological type, menopausal status and age at menarche.

**Figure 4:**
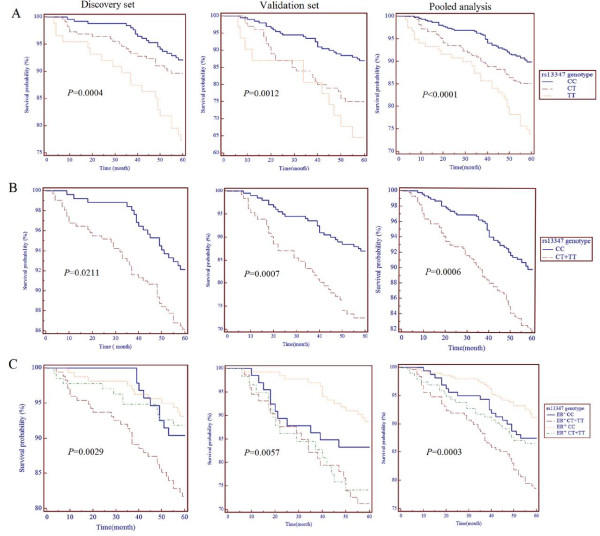
**Kaplan-Meier curves about survival probability in different rs13347C>T genotype carriers**. **(A) **difference in survival probability between CC, CT and TT carriers **(B) **difference in survival probability between CC and CT+TT carriers **(C) **difference in survival probability between ER^+^CC, ER^+^CT+TT, ER^-^CC and ER^-^CT+TT carriers.

The rs13347C > T polymorphism was further tested in the validation set. In this dataset, when compared with the rs13347CC genotype, the CT and TT genotypes were associated with poor survival (adjusted HR = 2.104, 3.144 and *P *= 0.0081, 0.015, respectively) and rs13347 CT+TT genotypes had a 2.34-fold increased death risk (*P *= 0.0010). Also, in the pooled analysis of the two cohorts we found that the rs13347 CT or rs13347 TT genotype had a 1.54-fold or 2.84-fold increased death risk (*P *= 0.00378 and *P *< 0.001) and the HR is 1.873 (*P *= 0.0007) for the CT+TT carriers (Table 2). As is also shown in Figure 4A, B, CT or TT carriers have lower survival probability in discovery set, validation set and pooled analysis. The contribution of interaction between rs13347 variation and ER status to a five-year survival rate of breast cancer patients was further investigated and it was found that ER negative T carriers yield the lowest survival probability (Figure 4C). However, no significant contribution was found in the other four polymorphisms.

## Discussion

Associations between breast cancer susceptibility and *CD44 *polymorphisms have not been detected in any population using case-control studies. In this molecular epidemiological study we sought to identify genetic factors that confer individual susceptibility to breast cancer. Our results obtained by analyzing 1,853 breast cancer patients and 1,992 controls from two study centers showed that the functional variation rs13347 T in the *CD44 *was associated with increased risk for developing breast cancer and yields lower five-year survival probability. However, there exists no significant difference in the susceptibility and prognosis affect to breast cancer between different genotypes of the other four polymorphisms.

CD44 is a ubiquitously expressed family of cell adhesion glycoproteins comprising an N-terminal extracellular domain, a membrane proximal region, a transmembrane domain and a cytoplasmic tail. The family is coded by the human *CD44 *gene, which is mapped to chromosomal locus 11p13 and is composed of two groups of exons [27]. Exons 1 to 5 and 16 to 20 are spliced together to form a transcript encoding the ubiquitously expressed standard isoform (CD44s). The variable exons 6 to 5 (known as v1 to 10) can be alternatively spliced and inserted to the standard form between exons 5 and 16 [28]. The multiple functions of the CD44 family are generated by their binding of HA (hyaluronic acid) and some other extracellular molecules [28]. CD44 regulates breast cancer through several mechanisms. Interaction of hyaluronan and CD44 can promote breast cancer cell adhesion and inhibited invasion [29]. Besides, binding of hyaluronan to CD44v3 can stimulate breast cancer cell growth, survival and invasion through the Rho and PI3K-AKT signaling pathways [30]. Moreover, the migration of metastatic breast cancer cells can be increased by the interaction of CD44v3, 8 to 10 with ankyrin promoted by Rho kinase [31]. Based on the above, it is reasonable to predict that changes in the expression or function of CD44 will play a pivotal role in the development and progression of breast cancer. Krech R. *et al. *reported a significant increase in the CD44 expression in breast cancer compared to normal breast epithelium [18]. These findings correspond with our results that *CD44 *rs13347 T carriers possess higher protein levels and, therefore, they are more susceptible to breast cancer and have poorer prognosis.

Much interest has been generated by the recent discovery that CD44 is a surface marker of BCICs [9]. Lin *et al. *found that CD44^pos^CD24^neg ^and CD44^pos^CD24^pos^cell populations in estrogen receptor (ER) α-negative breast tumors are tumorigenic in murine xenograft models, which indicate CD44 as a hallmark of BCIC in ER-negative breast cancer [32]. Similarly, in a study examining the expression profile of cancer stem cell markers in eight human breast cancer cell lines, Lee *et al. *found that CD44 was expressed mostly in basal-like cell lines, including MDA-MB-468, MDA-MB-231 and HCC1937, which were all ER negative [33]. Recently, substantial progress has been made in the identification of BCICs and there is accumulating evidence that these cells might be targets for transformation during mammary carcinogenesis [9]. Since CD44 contributes much to BCICs' maintenance and activity as its surface marker and BCICs play an important role in breast cancer tumorigenesis, it is inferable that the possible quantitative change of CD44 caused by rs13347 C/T mutation will affect breast cancer development, especially in ER-negative patients. In addition, the expression of ER also has important prognostic implications; that is, ER-positive tumors have a better prognosis in terms of overall survival, while ER-negative tumors have a more aggressive phenotype and poorer survival probability [34-36]. Although the exact mechanism is still unclear, there will be no doubt that some risk factor will do more for breast cancer generation, development and prognosis in ER-negative patients. These previous study results and inferences are consistent with our findings that the parlous role of rs13347 CT+TT is more pronounced in ER-negative patients and ER negative rs13347 T allele-carrying patients yield the minimum survival probability.

Although we have found that *CD44 *rs13347 variant genotypes (CT+TT) were associated with increased risk for breast cancer, our study may have certain limitations caused by the study design. For example, selection bias and/or systematic error may occur because the cases were from the hospital and the controls were from the community. Selection bias is a particular problem inherent in case-control studies, where it gives rise to non-comparability between cases and controls. In case-control studies, controls should be drawn from the same population as the cases, so they are representative of the population which produced the cases. In our present study, cases and controls in each center were collected from the same place during the same time and the breast cancer patient samples in our study were sporadic cancer patients, reducing the probability of selection bias from the maximum extent. Moreover, the fact that we have achieved a more than 95% study power (two-sided test, α = 0.05) to detect an OR of 1.72 for the rs13347 CT+TT genotypes, which occurred at a frequency of 42.5% in the controls, compared with the rs13347 CC genotype, suggesting that this finding is noteworthy.

## Conclusions

Our study indicated that compared with the *CD44 *rs13347 CC genotype, the variant genotypes (CT+TT) can elevate the risk of breast cancer and predicts poorer five-year survival rate in both Southern and Eastern Chinese populations. Moreover, the phenomenon is more obvious in ER-negative breast cancer patients. To our best knowledge, our study first demonstrated a significant association between the *CD44 *rs13347 C/T polymorphism and risk of breast cancer. Moreover, larger, preferably population-based case-control studies, as well as well-designed mechanistic studies, are warranted to validate our findings in Chinese populations or to investigate the association between this polymorphism with different tumors in different ethnicities.

## Abbreviations

BCICs: breast cancer-initiating cells; BMI: body mass index; CHB: Chinese Han Beijing; CIC: cancer initiating cell; ER: estrogen receptor; HR: hazard ratio; HWE: Hardy-Weinberg equilibrium; LD: linkage disequilibrium; MAF: minor allele frequency; MALDI-TOF: Matrix Assisted Laser Desorption Ionization-Time of Flight; OR: odds ratio; PR: progesterone receptor; SNP: single nucleotide polymorphism; UTR: untenslated region.

## Competing interests

The authors indicated no potential conflicts of interests.

## Authors' contributions

YZ and LJ conceived the idea for the present analysis and designed the study. XZ provided the study material. JD, JZ, YY and NL collected the data. LJ, JL and HW analyzed and interpreted the data. YZ, LJ and JD prepared the manuscript. All authors revised the manuscript and gave their final approval.

## Supplementary Material

Additional file 1 **Distributions of characteristics among breast cancer patients and controls in Chinese populations used for association study**. Age, age at menarche, body mass index, family history, pathological type, stage, estrogen receptor status and progesterone receptor status distributions among breast cancer patients and healthy controls from Suzhou and Guangzhou center. Click here for file

Additional file 2 **Demographic and clinical characteristics of breast cancer patients in the five-year survival discovery and validation sets**. Age, age at menarche, body mass index, family history, pathological type, stage, estrogen receptor status and progesterone receptor status distributions among the patients and healthy controls used for five-year survival analysis from Suzhou and Guangzhou center. Click here for file

Additional file 3 **Haplotype block analysis of polymorphisms in CD44 gene**. Six potential functional SNPs (minor allele frequency > 5%) were used to analyze the haplotype block based on the CHB (Chinese Han Beijing) population data of HapMap. Click here for file

Additional file 4 **Genotyping analysis of candidate SNPs**. The figure shows representative MALDI-TOF mass spectrometry profiles for different allelic PCR products containing the *CD44 *rs13347, rs10836347, rs1425802, rs11821102 and rs713330 polymorphism sites. Click here for file

Additional file 5 **Direct sequencing of candidate SNPs**. *CD44 *rs13347, rs10836347, rs1425802, rs11821102 and rs713330 genotyping by direct sequencing. Click here for file

Additional file 6 **Western blotting analysis in different rs13347 genotypes carriers**. Relative CD44 expression in 15 CC samples, 17 CT samples and 7 TT samples. Click here for file

Additional file 7 **Immunohistochemistry assay in different rs13347 genotypes carriers**. CD44 immunohistochemistry assay results in 15 CC samples, 12 CT samples and 4 TT samples. Click here for file
