# Supplementary material for: CD44 rs13347 C>T polymorphism predicts breast cancer risk and prognosis in Chinese populations
Source: Breast Cancer Res. 2012 Jul 12;14(4):R105. doi: 10.1186/bcr3225 (PMC3680922; doi:10.1186/bcr3225)
Supplement: Additional file 1 — Distributions of characteristics among breast cancer patients and controls in Chinese populations used for association study. Age, age at menarche, body mass index, family history, pathological type, stage, estrogen receptor status and progesterone receptor status distributions among breast cancer patients and healthy controls from Suzhou and Guangzhou center. [file bcr3225-S1.DOC]

**Supplementary Tab.1** Distributions of characteristics among breast cancer patients and controls in Chinese populations used for association study

| **characteristics** | Suzhou population | |  | Guangzhou population | |  | Overall | |
| --- | --- | --- | --- | --- | --- | --- | --- | --- |
|  | Case n (%) | Control n (%) |  | Case n (%) | Control n (%) |  | Case n (%) | Control n (%) |
| **Age(years)** |  |  |  |  |  |  |  |  |
| ≤40 | 224 (21.3) | 230 (19.9) |  | 206 (25.6) | 218 (26.1) |  | 430 (23.2) | 448 (22.5) |
| 41-60 | 626 (59.7) | 789 (68.2) |  | 488 (60.7) | 495 (59.3) |  | 1114 (60.1) | 1284 (64.5) |
| ≥60 | 199 (19.0) | 138 (11.9) |  | 110 (13.7) | 122 (14.6) |  | 309 (16.7) | 260 (13.0) |
| **Age at menarche(years)** |  |  |  |  |  |  |  |  |
| ≤14 | 555 (52.9) | 739 (63.9) |  | 423 (52.6) | 427 (51.1) |  | 978 (52.8) | 1166 (58.5) |
| >14 | 494 (47.1) | 418 (36.1) |  | 381 (47.4) | 408 (48.9) |  | 875 (47.2) | 826 (41.5) |
| **Body mass index** |  |  |  |  |  |  |  |  |
| ≤20 | 412 (39.3) | 167 (14.4) |  | 202 (25.1) | 123 (14.7) |  | 614 (33.1) | 290 (14.6) |
| 20-28 | 604 (57.6) | 877 (75.8) |  | 555 (69.0) | 610 (73.1) |  | 1159 (62.6) | 1487 (74.6) |
| ≥28 | 33 ( 3.1) | 113 ( 9.8) |  | 47 ( 5.9) | 102 (12.2) |  | 80 ( 4.3) | 215 (10.8) |
| **Family history** |  |  |  |  |  |  |  |  |
| Positive | 102(9.7) | 88 (7.6) |  | 81 (10.1) | 59 ( 7.1) |  | 183 (9.9) | 147 ( 7.4) |
| Negative | 947(90.3) | 1069(92.4) |  | 723 (89.9) | 776 (92.9) |  | 1670 (90.1) | 1845 (92.6) |
| **Pathological type** |  |  |  |  |  |  |  |  |
| Invasive ductal carcinoma | 896 (85.4) |  |  | 589 (73.3) |  |  | 1485 (80.1) |  |
| other carcinoma | 153 (14.6) |  |  | 215 (26.7) |  |  | 368 (19.9) |  |
| **Stage** |  |  |  |  |  |  |  |  |
| I | 282 (26.9) |  |  | 169 (21.0) |  |  | 451 (24.3) |  |
| II | 652 (62.2) |  |  | 454 (56.5) |  |  | 1106 (59.7) |  |
| III | 98 ( 9.3) |  |  | 108 (13.4) |  |  | 206 (11.1) |  |
| IV | 17 ( 1.6) |  |  | 73 ( 9.1) |  |  | 90 ( 4.9) |  |
| **Estrogen receptor status** |  |  |  |  |  |  |  |  |
| positive | 572 (54.5) |  |  | 462 (57.5) |  |  | 1034 (55.8) |  |
| negative | 477 (45.5) |  |  | 342 (42.5) |  |  | 819 (44.2) |  |
| **progesterone receptor status** |  |  |  |  |  |  |  |  |
| positive | 599 (57.1) |  |  | 484 (60.2) |  |  | 1083 (58.4) |  |
| negative | 450 (42.9) |  |  | 320 (39.8) |  |  | 770 (41.6) |  |
