# Supplementary material for: CD44 rs13347 C>T polymorphism predicts breast cancer risk and prognosis in Chinese populations
Source: Breast Cancer Res. 2012 Jul 12;14(4):R105. doi: 10.1186/bcr3225 (PMC3680922; doi:10.1186/bcr3225)
Supplement: Additional file 2 — Demographic and clinical characteristics of breast cancer patients in the five-year survival discovery and validation sets. Age, age at menarche, body mass index, family history, pathological type, stage, estrogen receptor status and progesterone receptor status distributions among the patients and healthy controls used for five-year survival analysis from Suzhou and Guangzhou center. [file bcr3225-S2.DOC]

**Supplementary Tab.2** Demographic and clinical characteristics of breast cancer patients in the 5-year survival discovery and validation sets

| **Variables** |  | Suzhou Population (N=566) | |  | Guangzhou Population (N=331) | |  | Log-rank |  | HR(95%CI) *^+^ |
| --- | --- | --- | --- | --- | --- | --- | --- | --- | --- | --- |
|  |  | Breast cancer patients | Deaths |  | Breast cancer patients | Deaths |  |  |  |  |
|  |  | n (%) | (n= 63) |  | n (%) | (n= 62) |  | *P** |  |  |
| **Ages(years)** |  |  |  |  |  |  |  |  |  |  |
| ≤47 |  | 304 (53.7) | 38 |  | 166 (50.2) | 24 |  | 0.481 |  | 1.00 (Reference) |
| >47 |  | 262 (46.3) | 25 |  | 165 (49.8) | 38 |  |  |  | 0.88 (0.62-1.25) |
| **Age at menarche(years)** |  |  |  |  |  |  |  |  |  |  |
| ≤15 |  | 403 (71.2) | 45 |  | 230 (69.5) | 39 |  | 0.36 |  | 1.00 (Reference) |
| >15 |  | 163 (28.8) | 18 |  | 101 (30.5) | 23 |  |  |  | 1.19 (0.82-1.73) |
| **Menstrual history** |  |  |  |  |  |  |  |  |  |  |
| Premenopause |  | 399 (70.5) | 46 |  | 160 (48.3) | 27 |  | 0.322 |  | 1.00 (Reference) |
| Menopause |  | 167 (29.5) | 17 |  | 171 (51.7) | 35 |  |  |  | 1.20 (0.84-1.71) |
| **Body mass index** |  |  |  |  |  |  |  |  |  |  |
| ≤20 |  | 207 (36.6) | 24 |  | 71 (21.5) | 13 |  | 0.409 |  | 1.00 (Reference) |
| 20-28 |  | 345 (60.9) | 37 |  | 234 (70.7) | 42 |  |  |  | 1.02 (0.69-1.51) |
| ≥28 |  | 14 (2.5) | 2 |  | 26 (7.8) | 7 |  |  |  | 1.79 (0.86-3.71) |
| **Family history** |  |  |  |  |  |  |  |  |  |  |
| Positive |  | 50 (8.9) | 6 |  | 31 (9.4) | 4 |  | 0.757 |  | 1.00 (Reference) |
| Negative |  | 516 (91.1) | 57 |  | 300 (90.6) | 58 |  |  |  | 0.89 (0.41-1.90) |
| **Pathological type** |  |  |  |  |  |  |  |  |  |  |
| Invasive ductal carcinoma |  | 473 (83.6) | 48 |  | 203 (61.3) | 35 |  | 0.010 |  | 1.00 (Reference) |
| Other carcinoma |  | 93 (16.4) | 15 |  | 128 (38.7) | 27 |  |  |  | 1.63 (1.12-2.36) |
| **Stage** |  |  |  |  |  |  |  |  |  |  |
| I+II |  | 464 (82.0) | 18 |  | 207 (62.5) | 9 |  | <0.0001 |  | 1.00 (Reference) |
| III+IV |  | 102 (18.0) | 45 |  | 124 (37.5) | 53 |  |  |  | 14.66 (9.56-22.49) |
| **Estrogen receptor status** |  |  |  |  |  |  |  |  |  |  |
| Negative |  | 269 (47.5) | 41 |  | 139 (42.0) | 32 |  | 0.0017 |  | 1.00 (Reference) |
| Positive |  | 297 (52.5*)* | 22 |  | 192 (58.0) | 30 |  |  |  | 0.57 (0.40-0.81) |
| **Progesterone receptor status** |  |  |  |  |  |  |  |  |  |  |
| Negative |  | 242 (42.8) | 41 |  | 133 (40.2) | 27 |  | 0.002 |  | 1.00 (Reference) |
| Positive |  | 324 (57.2) | 22 |  | 198 (59.8) | 35 |  |  |  | 0.58 (0.41-0.82) |

Abbreviations: HR, hazard ratio;

*Data are for the combined discovery and validation sets.

^+^ Data was calculated by univariate cox regression analysis.
