# Supplementary figures and images for: CD44 rs13347 C>T polymorphism predicts breast cancer risk and prognosis in Chinese populations
Source: Breast Cancer Res. 2012 Jul 12;14(4):R105. doi: 10.1186/bcr3225 (PMC3680922; doi:10.1186/bcr3225)

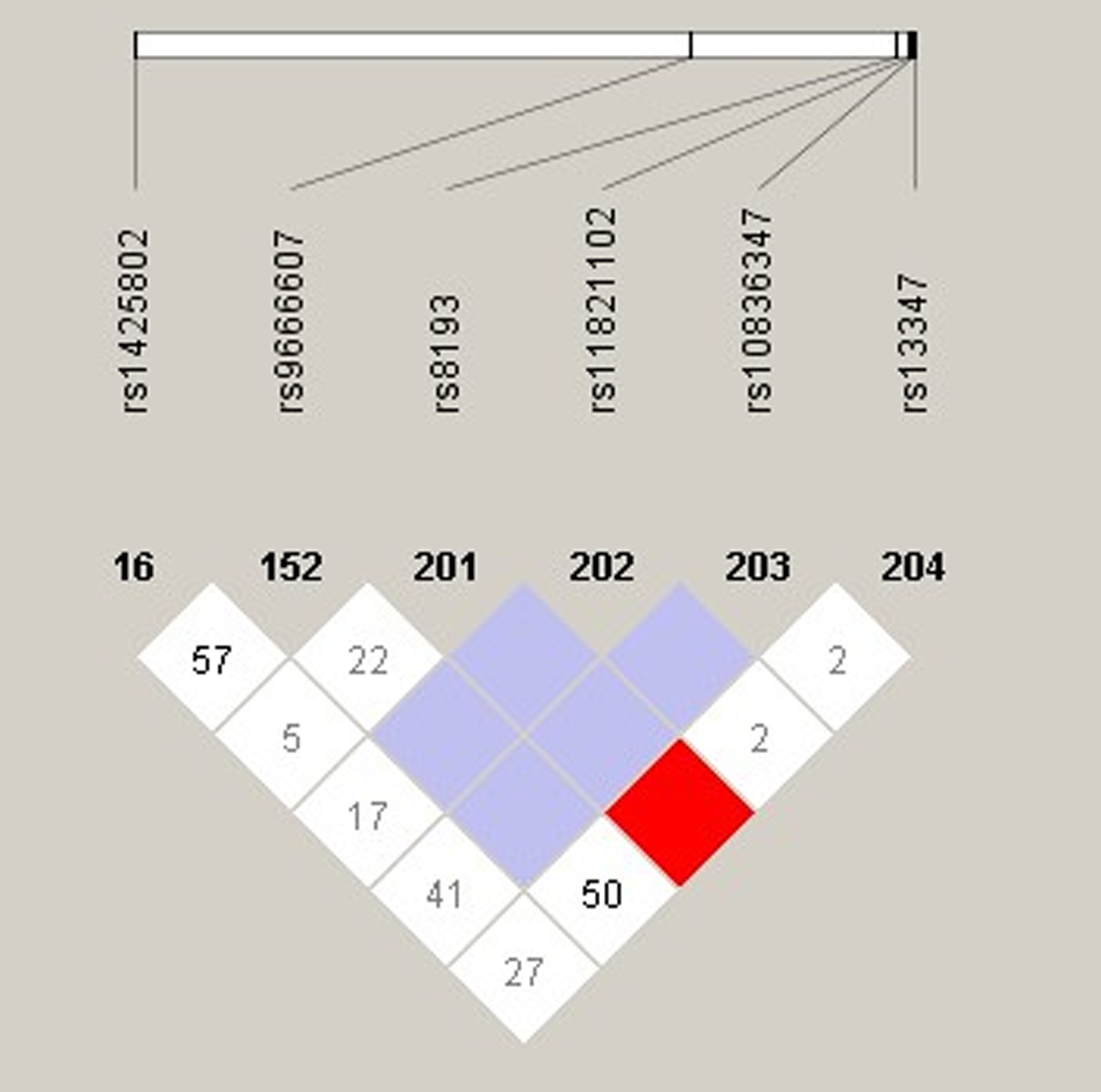

Supplement: Additional file 3 — Haplotype block analysis of polymorphisms in CD44 gene. Six potential functional SNPs (minor allele frequency > 5%) were used to analyze the haplotype block based on the CHB (Chinese Han Beijing) population data of HapMap. [file bcr3225-S3.TIFF]

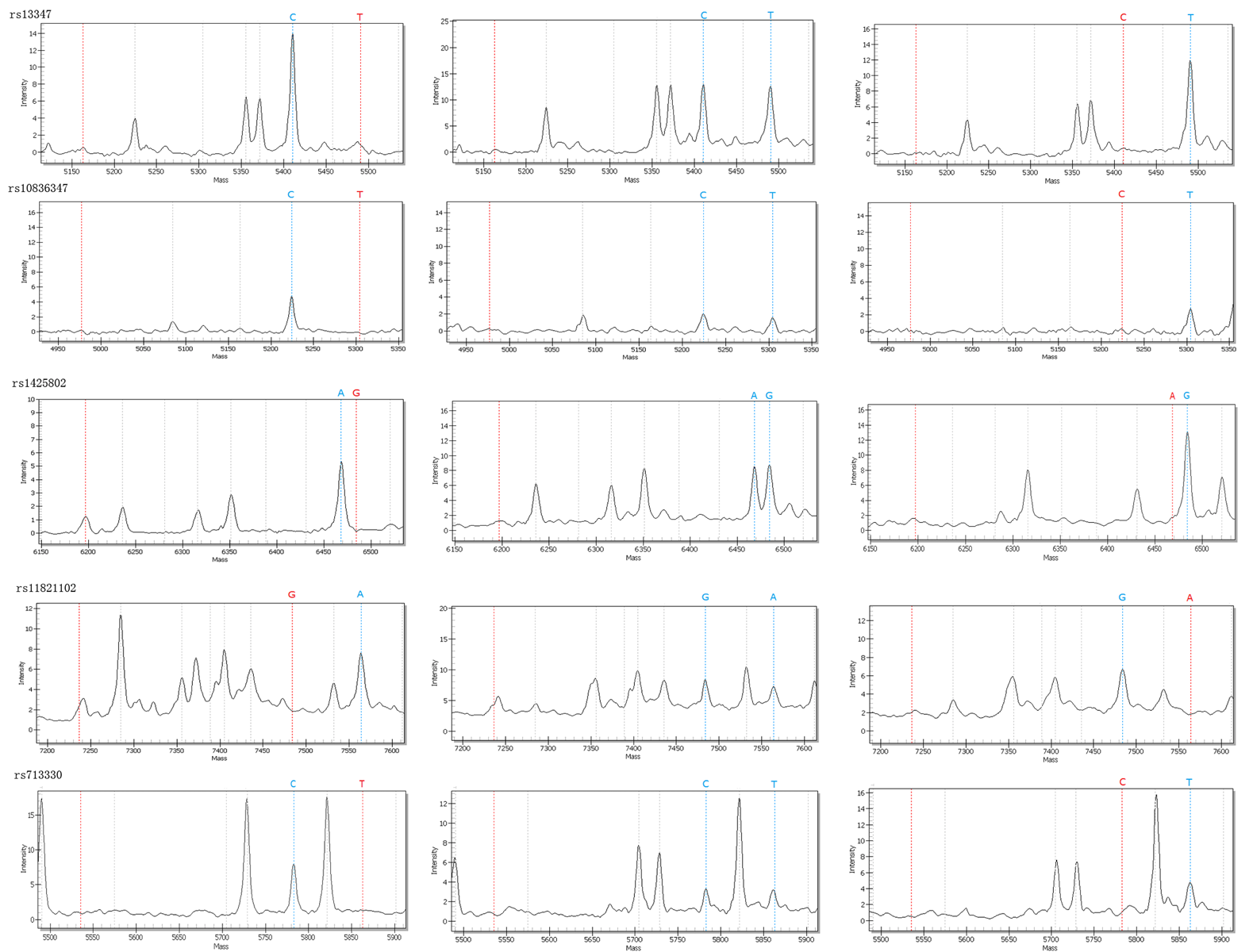

Supplement: Additional file 4 — Genotyping analysis of candidate SNPs. The figure shows representative MALDI-TOF mass spectrometry profiles for different allelic PCR products containing the CD44 rs13347, rs10836347, rs1425802, rs11821102 and rs713330 polymorphism sites. [file bcr3225-S4.TIFF]

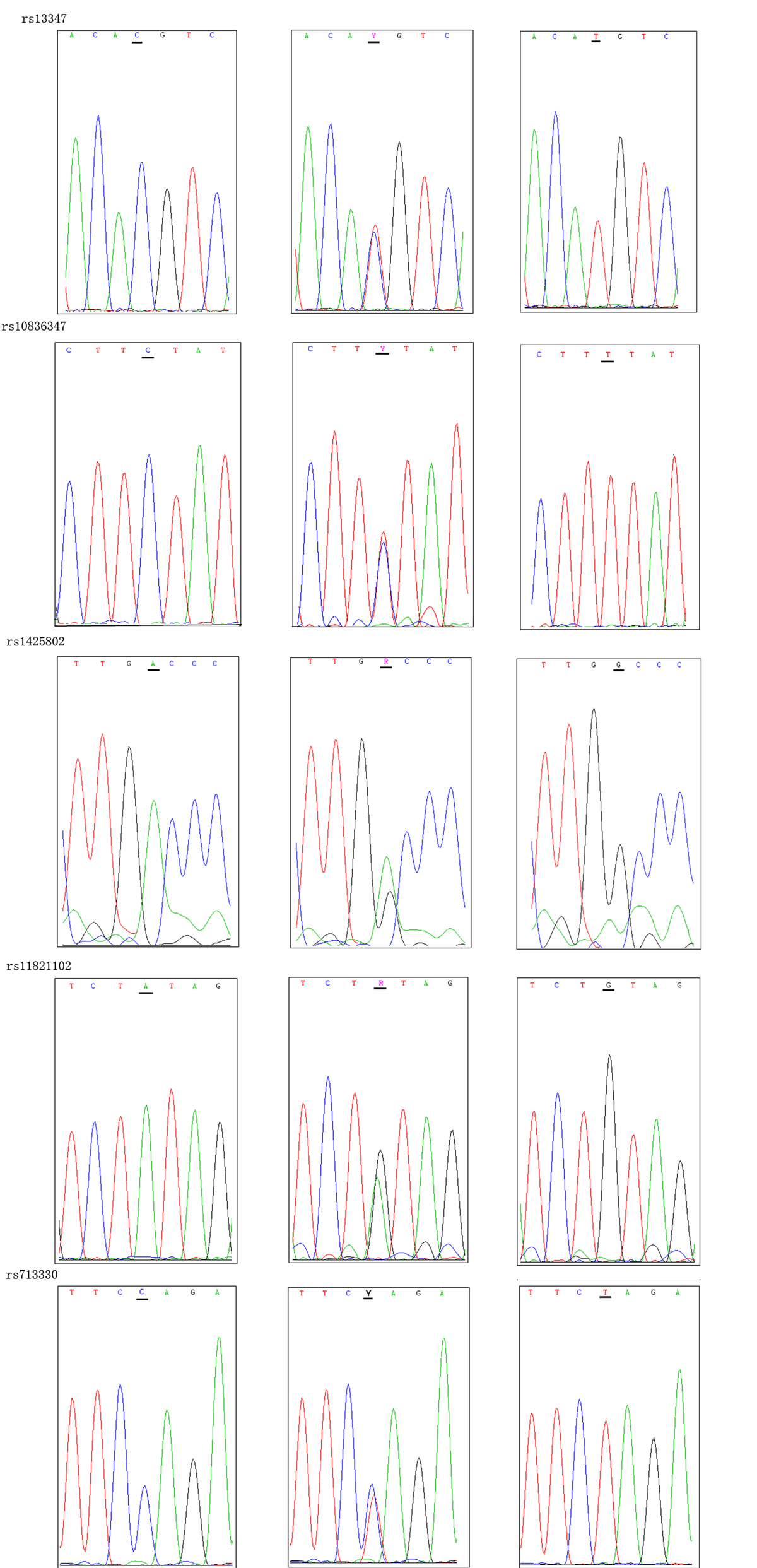

Supplement: Additional file 5 — Direct sequencing of candidate SNPs. CD44 rs13347, rs10836347, rs1425802, rs11821102 and rs713330 genotyping by direct sequencing. [file bcr3225-S5.TIFF]
