# Supplementary material for: CD44 rs13347 C>T polymorphism predicts breast cancer risk and prognosis in Chinese populations
Source: Breast Cancer Res. 2012 Jul 12;14(4):R105. doi: 10.1186/bcr3225 (PMC3680922; doi:10.1186/bcr3225)
Supplement: Additional file 6 — Western blotting analysis in different rs13347 genotypes carriers. Relative CD44 expression in 15 CC samples, 17 CT samples and 7 TT samples. [file bcr3225-S6.DOC]

**Supplementary Tab.3**.Results of Western blotting in different genotypes

| CC | CT | TT |
| --- | --- | --- |
| 0.1301 | 0.7716 | 0.8387 |
| 0.3241 | 0.4760 | 0.7375 |
| 0.2745 | 0.1857 | 0.9405 |
| 0.2151 | 0.7787 | 0.8935 |
| 0.3438 | 0.5516 | 0.6692 |
| 0.3081 | 0.6829 | 0.7558 |
| 0.3041 | 0.4426 | 1.0308 |
| 0.2865 | 0.8709 |  |
| 0.1940 | 0.2045 |  |
| 0.2492 | 0.3041 |  |
| 0.1448 | 0.1040 |  |
| 0.1823 | 0.1503 |  |
| 0.2588 | 0.2356 |  |
| 0.1529 | 0.4123 |  |
| 0.2160 | 0.4352 |  |
|  | 0.5878 |  |
|  | 0.7101 |  |
