# Supplementary material for: CD44 rs13347 C>T polymorphism predicts breast cancer risk and prognosis in Chinese populations
Source: Breast Cancer Res. 2012 Jul 12;14(4):R105. doi: 10.1186/bcr3225 (PMC3680922; doi:10.1186/bcr3225)
Supplement: Additional file 7 — Immunohistochemistry assay in different rs13347 genotypes carriers. CD44 immunohistochemistry assay results in 15 CC samples, 12 CT samples and 4 TT samples. [file bcr3225-S7.DOC]

**Supplementary Tab. 4** Results of immunohistochemistry assay in different genotypes

|  | 0-20% | 21-40% | 41-60% | 61-80% | >81% |
| --- | --- | --- | --- | --- | --- |
| CC (n=15) | 3 | 7 | 4 | 1 |  |
| CT (n=12) | 1 | 3 | 5 | 2 | 1 |
| TT (n=4) |  |  | 1 | 1 | 2 |
